# Design and development of an affordable multi-mode small animal ventilator

**DOI:** 10.1038/s41598-025-28241-w

**Published:** 2025-11-18

**Authors:** Patryk Dzierzawski, Bernd Flamm, Verena Hegele, Sashko Spassov, Christin Wenzel, Stefan Schumann, Sara Lozano-Zahonero

**Affiliations:** Department of Anaesthesiology and Critical Care, Medical Center-University of Freiburg, Faculty of Medicine, 79106 Freiburg, Germany

**Keywords:** Mechanical ventilation, Small animal ventilation, Customized ventilation profiles, Biomedical engineering, Preclinical research

## Abstract

Small animal experiments are essential in biomedical research, particularly for preclinical investigations. These experiments frequently require mechanical ventilation, but the market offers expensive and functionally limited ventilators. To address this, we developed a cost-effective multi-mode ventilator using commercially available components. Our ventilator utilizes a microcontroller as the primary processing unit, receiving settings from a computer interface. The microcontroller synchronizes five valves to control inspiration and expiration of breathing cycles while managing airflow via piston pumps to generate the required tidal volume. This ensures precise breath regulation in terms of controlling the desired pressure-volume schematic in small animal respiratory systems. Positive end-expiratory pressure is manually adjustable. The system emulates conventional profiles like Volume Control Ventilation and Pressure Control Ventilation, while offering customizable inspiration and expiration patterns (sinusoidal, linear, and exponential). Operating specifications include tidal volumes of 1–15 ml and respiratory rates up to 120 breaths per minute. This versatile system provides customizable ventilation profiles with precise inspiration-expiration cycle synchronization, enabling tailored experimental conditions. Its cost-effectiveness makes it accessible to a broader range of researchers. This system marks a significant advancement in small animal research by offering precise and flexible ventilation strategies that enhance experimental accuracy and contribute to improved research outcomes.

## Introduction

Small animal experiments are essential in preclinical research, providing a foundation for investigating new drugs, therapeutic approaches, and medical devices before human testing. These models enable researchers to assess the safety and efficacy of interventions before advancing to more complex and costly preclinical trials involving large animal models or clinical trials involving human subjects. Small animal ventilators play a crucial role in these studies, supporting the animals’ respiratory function and maintaining physiological parameters. However, commercially available ventilators for small animals are expensive and functionally limited. They often lack flexibility in defining custom ventilation patterns, do not support advanced features such as controlled expiration^1^ or recruitment maneuvers, and their proprietary designs make modification or maintenance difficult. To address these limitations, there is a need for a small animal ventilator capable of performing standard ventilation modes, such as Volume Control Ventilation (VCV) and Pressure Control Ventilation (PCV), with a high degree of flexibility in parameter settings and the capability to define custom ventilation patterns. Therefore, we have developed a ventilator system with a customizable ventilation setup. The material costs for the developed ventilator amount to approximately $800, making it significantly more affordable compared to commercially available devices. In addition, the modular design ensures robust operation with respect to reliability, reproducibility, and ease of maintenance, allowing components to be easily replaced or extended as needed. Therefore, the ventilator design has the potential to provide significant benefit for the preclinical research community, particularly those studying small animals such as rats^2–4^ or rabbits^5,6^.

## Methods

### Overview

**Fig. 1:**
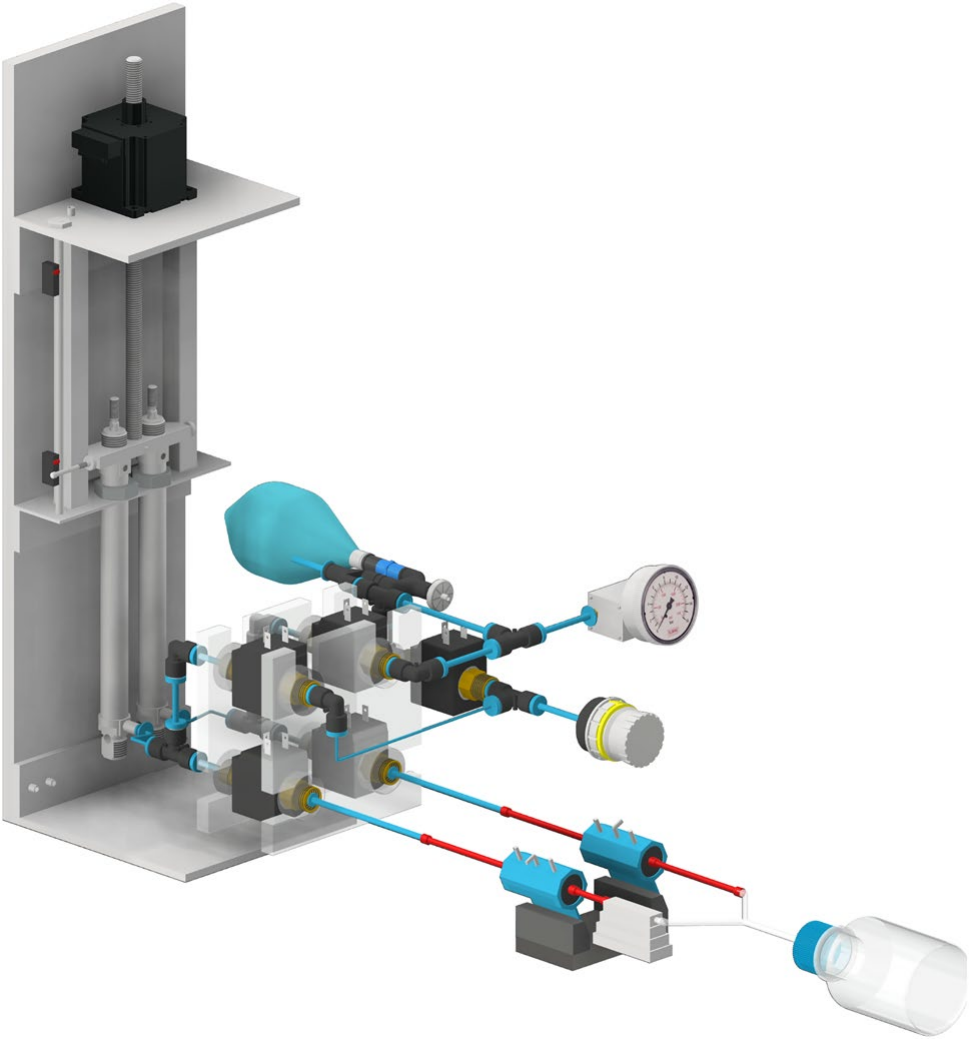
CAD model of the designed small animal ventilator.

**Fig. 2:**
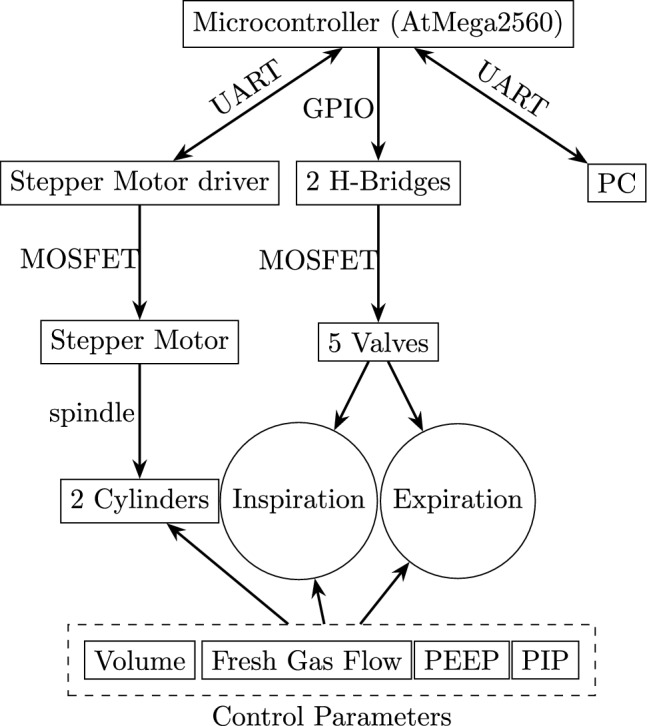
Basic schematic of the small animal ventilator with essential parts and inheritances.

The developed small animal ventilator (Fig. 1, Supplementary Fig. S1) incorporates a microcontroller as its primary processing unit. This microcontroller controls several system components (Fig. 2):

- Movements of the stepper motor, controlled by a dedicated stepper motor driver,
- Operation of five solenoid valves connected via two H-bridges,
- Adjustment of ventilation parameters via a computer connection using serial communication.

### Materials and technical setup

Historically, we began working with Arduino Sketch programming on the Arduino Mega 2560 Rev3 (Arduino S.r.l., Monza, Italy). So our microcontroller is the Atmel ATmega2560 (Atmel Corporation, San Jose, USA), a high-performance, low-power 8-bit microcontroller from the Atmel AVR series. It features four Universal Asynchronous Receiver Transmitters (UART), a byte-oriented two-Wire Serial Interface ($\text {I}^{2}$C), and 86 general-purpose I/O lines (GPIO), among other capabilities. We programmed and debugged the microcontroller using the Microchip Studio IDE for AVR and SAM devices, in conjunction with the Microchip Atmel-ICE (Microchip Technology Inc, Chandler, USA) through the JTAG interface. Communication with the stepper motor driver, specifically the Pololu Tic 36v4 (Pololu, Las Vegas, USA), occurs via UART. This driver operates the Nanotec non-captive linear actuator (Nanotec Electronic GmbH & Co. KG, Feldkirchen, Germany), which uses time-precise signals for each of the four stepper motor wires to control movement through the use of discrete MOSFETs. The bipolar stepper motor is connected to two cylinders (Festo GmbH & Co. KG, Esslingen, Germany) via a spindle, enabling fine volume control for inspiration and expiration. To be capable of controlling the whole breathing cycle, one cylinder manages preload and application of fresh inspiration gas, and the other manages take up and discharge of exhalation gas. To establish oxygen concentration of inspiration gas ranging from 21% to 100%, we integrated a fresh gas flow system comprising an air and oxygen mixer, a clinical standard wall airway connection, and a flow limiter (all Siemens-Elema AB, Göteborg, Sweden) in the inspiration airway. Additionally, for setting positive end-expiratory pressure (PEEP) we implemented a PEEP valve (Dräger GmbH, Lübeck, Germany) in the inspiration airway path. Moreover, to ensure pressure stability in the high-pressure range of the peak inspiratory pressure (PIP), we utilized a PIP valve (Dräger GmbH, Lübeck, Germany). To manage both pressure states and facilitate the volume movements between the cylinders and the animal, we employed BMV70303 valves (Bavaria Fluid Systems GmbH, Pforzheim, Germany). These are 2/2-way solenoid valves with coaxial flow and an inner diameter of 4 mm, designed to minimize flow and pressure interferences. Flow measurements were facilitated by two pneumotachographs (Fleisch 000, Dr. Fenyves und Gut Deutschland GmbH, Rangendingen, Germany), one in the inspiratory and the other in the expiratory airway branch. The pressure was measured by piezoelectric pressure sensors (SI - special instruments GmbH, Nördlingen, Germany). Both were connected through a data acquisition (DAQ) system (Model 779675-01, National Instruments, Austin, USA) to a PC by which the data was visualized and recorded (LabVIEW, National Instruments, Austin, USA) with a high sampling rate of 500Hz, ensuring precise and reliable results. The sensors were calibrated before measurements, with the pressure sensor calibrated using an external pressure generator. Further, the flow sensors were calibrated via an automatic calibration routine implemented in our small animal ventilator by shifting a defined volume. All components used are summarized in Supplementary Table S1, and a detailed circuit diagram is provided in Supplementary Fig. S2.

### Functionality and pneumatic schematic

**Fig. 3:**
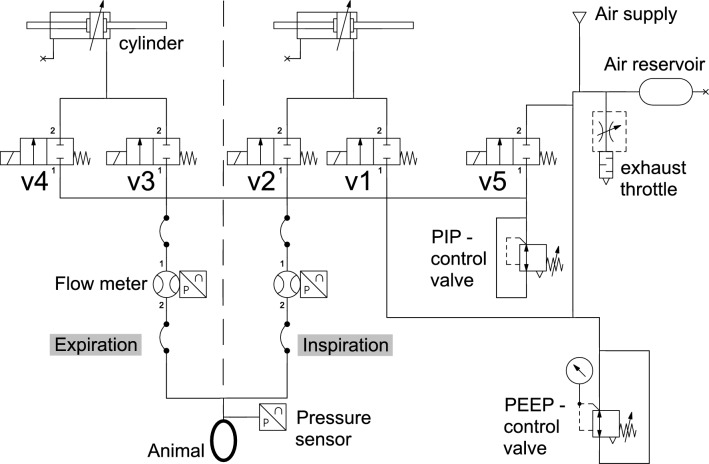
Pneumatic schematic of the small animal ventilator.

The pneumatic schematic in Fig. 3 and Supplementary Fig. S3 illustrates the interplay of components in our system, highlighting how cylinders, valves, and gas flow pathways work together to enable precise and controlled ventilation in small animals. It is to be noted that the two cylinders managing inspiration and expiration gas are driven synchronously to ensure that the same volume is removed during expiration as applied during inspiration. PEEP can be set at ranges from 0 to 15 $\text {cmH}_{2}$O, regulated by a PEEP valve. To ensure proper PIP during the ventilation, a PIP valve is connected to the expiration airway path.

#### Volume shift

This two-cylinder configuration ensures that the hysteresis in motor movement, lead screw mechanics, and positioning affects both inspiration and expiration in the same fashion. By utilizing one stepper motor and one lead screw, we can efficiently manage the system’s states - such as actual position, position calculations, commands, and communication - using a single microcontroller. This eliminates the need to compare results from two separate stepper motors or drivers. In our setup, we have used two Festo DSNU-16-125-PPV-A cylinders, which have a diameter of 16 mm and a stroke length of 125 mm. The theoretical maximum volume that this cylinder can generate is 25 ml. Off note, the Festo DSNU series would allow for easy replacement with cylinders of different diameters but maintaining the same stroke length of 125 mm (diameters ranging from 8 to 25 mm). To reduce the compressible volume in the tubing system, the cylinder’s working range is supposed to be between the most forward position and a point just before their maximum backward position. The motor utilized in our system is the Nanotec LA561S20-B-UQKE, which, in conjunction with the lead screw, functions as a linear actuator coupled to the cylinder. This setup allows for a precise volume displacement into and out of the animal. The stepper motor provides a resolution of 50.8 µm per step, with a maximum speed of 150 mm/s. By using half steps for more accurate movements, our resolution improves to 25.4 µm per step, corresponding to a volume displacement of 5.1 µl per step. Thus, our tidal volume conversion factor for the 16 mm diameter cylinder is 195.81, which indicates that 195.81 steps are required to move 1 ml of air volume.

#### Inspiration and expiration cycle

**Fig. 4:**
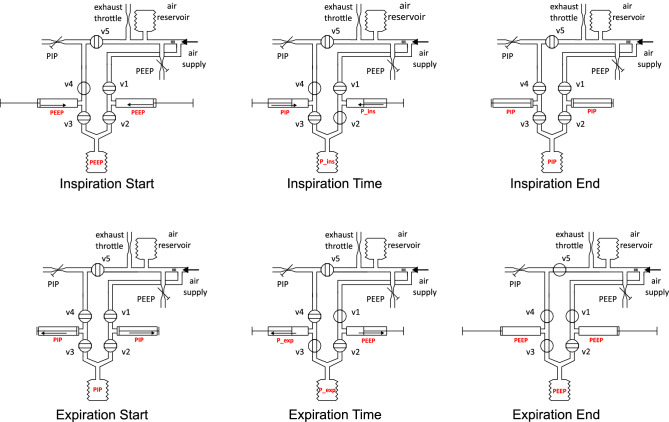
Breath cycle schematics illustrating valve states, pressure, and volume dynamics. This figure presents six schematics representing the sequential phases of a complete respiratory cycle generated by the small-animal ventilator. The phases include: the start of inspiration, inspiration phase, end of inspiration, start of expiration, expiration phase, and end of expiration. For clarity, sensor elements were omitted; the pressure sensor is located close to the animal, and two flow sensors (inspiratory and expiratory) are positioned immediately before the Y-piece.

The schematic (Fig. 4) illustrates the system’s components and their interactions during the inspiration and expiration cycles, divided into six distinct breath phases. Our system uses five valves: two in the inspiratory and two in the expiratory limb of the breathing circuit. And a fifth valve to enable passive expiration against PEEP pressure. Inspiratory valve v1 connects and disconnects fresh gas supply from the inspiratory piston. Inspiratory valve v2 is placed between the inspiratory piston and the Y-piece, which is the connection to the animal. Expiratory valve v3 is placed between the expiratory piston and the Y-piece. Expiratory valve v4 connects and disconnects PIP pressure to the expiratory piston. Expiratory valve v5 connects and disconnects PEEP pressure to the expiratory piston.

A mechanically adjustable PEEP valve is integrated into the inspiratory supply line and defines a constant pressure level used for both inspiratory pre-pressurization and end-expiratory stabilization. During expiration, valve v5 connects this regulated PEEP line to the expiratory limb, ensuring the target PEEP is maintained after exhalation. In contrast, PIP represents the peak inspiratory pressure generated by the forward movement of the inspiratory piston. Pre-pressurizing the inspiratory limb to the PEEP level before inspiration prevents an initial pressure drop when valve v2 opens.

*Inspiration start:* Before the beginning of the inspiration cycle, the motor and cylinders are retracted from the tip to achieve the desired volume. At this point, all valves except v4 are closed. The inspiratory cylinder and the space between valves v1 and v2 are filled with fresh air gas at PEEP pressure, since the system has already established PEEP during the homing sequence. Note homing sequence: valves v2 and v3 are closed, while v1 and v4 remain open, pressurizing the inspiratory cylinder to the defined PEEP level.

*Inspiration time:* The stepper motor moves forward to deliver the preset tidal volume to the animal. Valve v2 opens, allowing gas to flow from the inspiratory piston through the Y-piece into the airway.

*Inspiration end:* At the end of inspiration, valve v2 closes to retain the inspired volume within the animal. Simultaneously, valve v4 remains open to pressurize the expiratory limb to the PIP level. This prevents abrupt pressure shifts when the system transitions into expiration.

*Expiration start:* A very short time between the inspiration and expiration phase, all valves are closed, and the animal’s lungs are filled with the desired volume holding PIP pressure.

*Expiration time:* The stepper motor reverses direction, retracting the expiratory piston. Valve v3 opens to allow exhaled gas to enter the expiratory cylinder, while v1 opens to refill the inspiratory cylinder with fresh gas at PEEP pressure.

*Expiration end:* Shortly before the end of expiration, valves v4 and v5 open. Valve v5 connects the PEEP line to the expiratory limb, equilibrating the system at the set end-expiratory pressure. The PEEP valve acts as a back-pressure regulator, while the exhaust throttle provides passive flow resistance. Once equilibrium is reached, there is no pressure gradient, so gas movement ceases even though v3 remains open. The throttle’s constant resistance allows controlled gas release while maintaining stable PEEP.

The system’s air reservoir serves to smooth flow pulsations from the air supply, ensuring a consistent and reproducible gas volume delivery. It also provides an additional buffer that helps prevent re-inhalation of exhaled gas in rare cases of unexpected valve behavior.

**Fig. 5:**
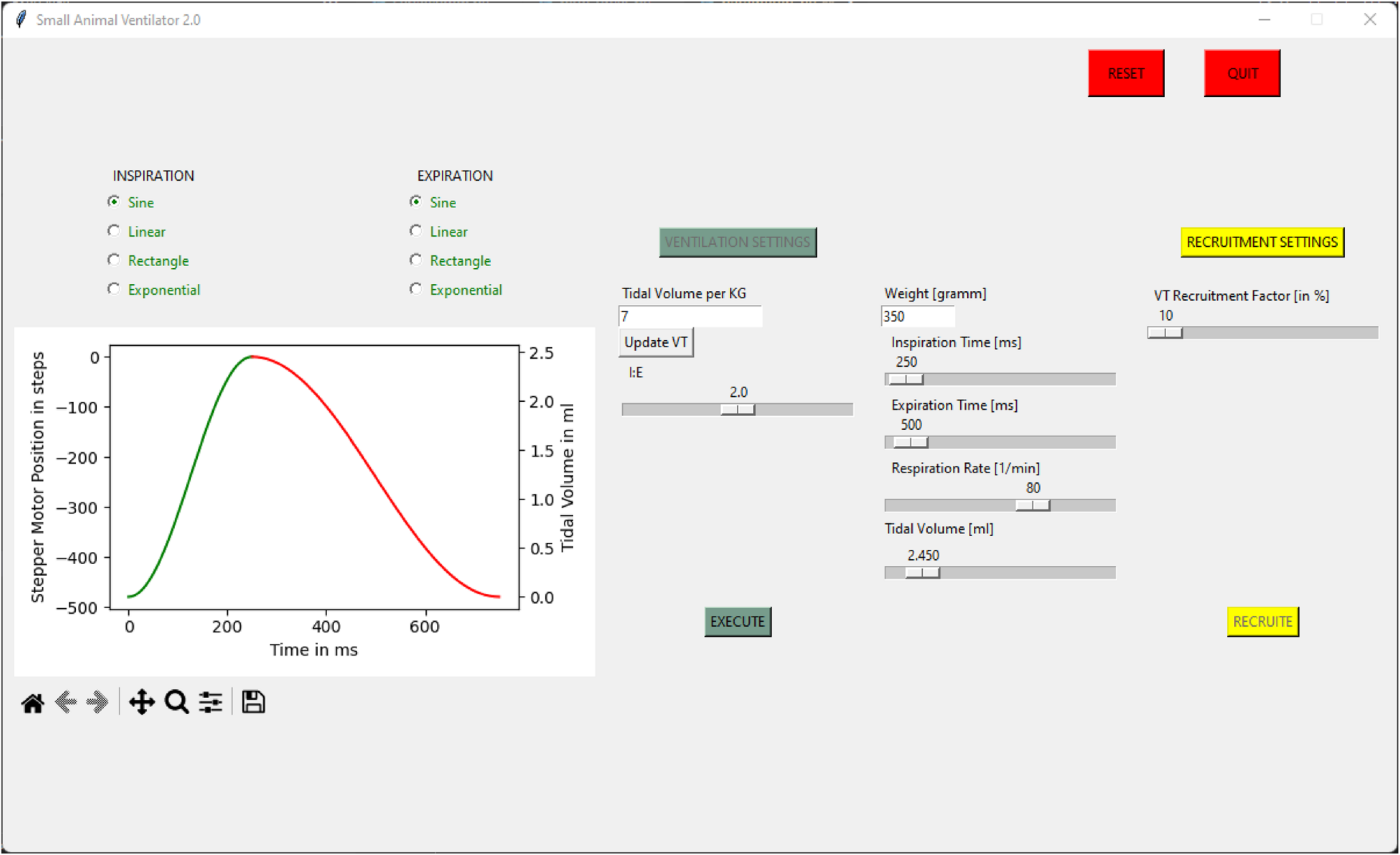
Graphical user interface for ventilator control and parameter adjustment. This figure displays the graphical user interface (GUI) developed for the small-animal ventilator system. The interface enables users to select from pre-configured inspiration and expiration flow profiles, ensuring rapid and reproducible configuration. Tidal volume is automatically calculated based on the animal’s body weight and the selected volume per kilogram. Key ventilation parameters–including respiratory rate, inspiration-to-expiration (I:E) ratio, and individual inspiratory and expiratory durations–can be freely adjusted via input fields or sliders. Additionally, a recruitment tidal volume factor, expressed as a percentage of the set tidal volume, allows users to temporarily increase tidal volume during recruitment maneuvers. Clearly labeled buttons initiate standard ventilation or recruitment cycles, and the layout ensures consistency across ventilation and recruitment settings.

Given the various choices available for each component - including the stepper motor, stepper motor driver, and cylinder - we conducted a thorough analysis of the system to guarantee the cylinder can move smoothly at any position and speed of the stepper motor at all times. To achieve this, the microcontroller continuously calculates the necessary current values as well as the maximum acceleration and deceleration parameters based on the current speed. The software in the Graphical User Interface (GUI), shown in Fig. 5, incorporates these limitations, ensuring only permissible settings can be made for the various pre-programmed ventilation profiles.

To maintain safety when adjusting settings such as the ventilation profile for inspiration and expiration, tidal volume, and respiratory rate, a validity check is performed in the GUI, and new parameter settings can only be applied (the button to confirm changes in parameter setting is either activated or deactivated) if they are valid. To ensure safety and reliable operation at all times, the motor movement was limited to 80% of the maximum speed. We tested various volume and breathing frequency settings across all profiles to determine the maximum speed within a profile. Testing confirmed that the motor may behave unpredictably between 80%-100% of the maximum motor speed due to the additional forces exerted by the attached cylinder, air displacement, and pressure differences within the system. This testing began with a tidal volume of 1 ml and inspiration and expiration times of 250 ms, increasing each by 1 ml until unusual behavior due to excessive maximum velocities was observed. Following this, we calculated a linear approximation of the relationship between tidal volume on the minimum of the inspiratory and expiratory times. This linear factor was multiplied by the tidal volume and then divided by the quotient of the minimal time and 100, which represents the maximum speed for 1 ml in 100 ms. We thoroughly tested all possible combinations of profiles and setting values, and all settings guarantee reliable motor performance.

#### Ventilation patterns

To drive the motor and, thereby, the cylinder, we calculate the velocity at each time point during a breath using dedicated formulas for sine, linear, and exponential profiles.

The linear and sinusoidal ventilation patterns allow smooth transitions between inspiration and expiration and promote homogeneous gas distribution, thereby improving lung recruitment and reducing ventilator-induced stress^7,8^. Furthermore, linear and sinusoidal ventilation support lung protection by preventing rapid pressure drops and maintaining mean airway pressure through a slower flow release^1,9^. However, a two-piston pump system was chosen because it provides greater flexibility and simplifies the programming of customized ventilation profiles, particularly for achieving the desired tidal volume and PEEP in small animals.

To ensure effective ventilator standard-setting options, we additionally employ recruitment maneuvers, which involve a sustained increase in airway pressure to open collapsed alveoli^10,11^. This is achieved by allowing an increase in airway pressure through a higher tidal volume. If activated in the GUI, the recruitment maneuver is executed once between two normal ventilation breaths in our system. For the recruitment standard pattern, we use a sine wave for both inspiration and expiration, each lasting 10 s. In the ventilation parameter settings, there is an option to recruit with a tidal volume that is 10% to 100% higher than the set ventilation volume. Users may choose a pre-configured pattern for the recruitment maneuver or implement a new one using a desired mathematical formula, similar to the ventilation profiles.

### Software and communication

To operate the microcontroller as the master and enable changes to ventilation profiles or parameters, we have established a standard serial communication using UART with the following settings: Baudrate 115200, no parity, one stop bit, and 8-bit data length (8$\mid$N$\mid$1). This allows any system capable of UART communication to easily modify the values, while the system is running.

The code continuously checks for incoming data on the UART channel, interprets commands, as depicted in Table 1, to update profiles and tidal volume values, and allows for system resets. Additionally, safety features are included to ensure secure operation by limiting the motor’s current, acceleration, and deceleration based on the current velocity and other relevant factors, as outlined earlier. The system also verifies motor movements by comparing target positions with the actual positions read by the motor driver, ensuring that the motor moves the cylinders the desired number of steps for a specific volume.

**Table 1:** UART commands for real-time adjustment of ventilator parameters during operation.

Command	Values	Function note
0xAF	uint8_t ins_profile, uint8_t exp_profile	Set inspiration profile and expiration profile
0xC2	uint16_t volume	Volume factorized by 1000 (send 3500 mean 3.500 ml)
0x9A	uint16_t inspiration_time	Set inspiration time
0xA5	uint16_t expiration_time	Set expiration time
0xDD	None	Reset by new homing

For example, to set a VCV profile with a tidal volume of 1.75 ml, an inspiratory time of 200 ms, and an expiratory time of 400 ms - which results in an Inspiration:Expiration (I:E) ratio of 1:2 and a respiratory rate of 100 breaths per minute - the data sent would look like this:


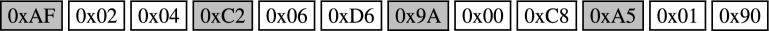


The command ’0xAF’ sets the profiles; the first byte is for the inspiration profile, and the second byte is for the expiratory profile. The profiles are encoded as follows: Sine (1), Linear (2), Rectangle (3), and Exponential (4). In this example, we have a linear inspiration flow and an exponential expiration flow. The tidal volume is set after the ’0xC2’ byte, sending two bytes as a 16-bit big-endian integer multiplied by 1000, allowing for precise milliliter settings with three decimal places. Therefore, ’0x06D6’ in big-endian format translates to 1750 in decimal, which is calculated on the microcontroller side as 1.75 ml. Setting the timing values follows a similar approach: here, ’0x00C8’ (200) for inspiratory time and ’0x0190’ (400) for expiratory time.

Another UART channel was programmed for debugging, using the same serial connection parameters to send unsigned 32-bit big-endian values, with the two frame start bytes ’0xAA’, ’0xFF’. The third UART communication interface is for the stepper motor driver. The connection settings are the same, although the baud rate is 115385, allowing the fastest possible communication supported by the Pololu Tic36v4. This establishes bi-directional communication, enabling us to send commands and values to the stepper motor driver and read data from it. The only values we read from the motor driver are the current position and current velocity. In each microcontroller loop cycle, we send essential updates to the stepper motor driver, specifically for the variables maxAccel, maxDecel, and most importantly, the function setVelocity() for moving the stepper motor. We opted not to use the motor driver’s built-in function goToPosition() due to the slow, jerky behavior it produces. Instead, we calculate the desired velocity as a factorized difference between the actual position and the target position, depending on the ventilation profile - considering both inspiration and expiration - as well as the volume, inspiration time, and expiration time. The only exception is the rectangular pattern, both in the inspiration and expiration. For this, we specifically use the goToPosition() function with the validated maximum speed, maximum acceleration, and deceleration in order to achieve the fastest possible change in position and, thus, a volume shift.

### System validation

To validate our system, we conducted a volume check to confirm that the set volume meets the cylinder’s output through the valves to the animal. The following values for ventilation apply to tests with the lung model presented in this manuscript:

- $\mathrm {V_T}$: 2.45 ml, e.g. 350 g animal weight, 7 ml per kilogram
- I:E-ratio: 1:2.0
- Respiratory rate: 80 breaths per minute
- IT: 250 ms, ET: 500 ms.

We used a lung model with constant compliance as a substitute, which is a common procedure in ventilation research^12–14^, as well as a small flexible test lung.

Animal experiments and related methods were approved and conducted in accordance with the guidelines of the local Animal Welfare Commission (Ethics Committee University of Freiburg, Germany, permissions No. G23/043). Housing and animal care procedures took place at the University Medical Center of Freiburg, Germany, and were in compliance with the European Directive 2010/63/EU. The authors confirm that all experiments were conducted in accordance with the ARRIVE guidelines^15^. To validate our system in in-vitro experiments, we used 15 male Sprague Dawley rats (Janvier Labs, Saint-Berthevin, France) with an average body weight of 386±34 g. Animals were anesthetized with 100 mg/kg ketamine and 1 mg/kg medetomidine intraperitoneally and placed on a heating pad to keep body temperature. A tracheotomy was established to facilitate mechanical ventilation. Before onset of mechanical ventilation, muscular relaxation was induced by intraperitoneal instillation of 1 mg/kg pancuronium. Thereafter, animals were monitored (blood pressure and ECG) and anesthesia was maintained by continuous administration of ketamine/midazolam/medetomidine (via catheterized femoral vein) and pancuronium, as described in^7^. Each animal underwent both VCV and sinusoidal ventilation in a cross-over design. We set the ventilation parameters to a fraction of inspired oxygen ($\mathrm {FiO_{2}}$) of 0.30, an I:E-ratio of 1:2, a $\mathrm {V_T}$ of 8 ml/kg, and PEEP of 4 cmH${_2}$O. We adjusted the respiratory rate to maintain the arterial partial pressure of carbon dioxide (paCO${_2}$) within the physiological range of 35-45 mmHg.

## Results

Our system successfully generated various common ventilation patterns. This includes standard profiles such as VCV, which provides a linear flow during inspiration and an exponential flow during expiration, and PCV, which is characterized by an exponential flow in both phases. For the latter, it should be noted that PCV is not a real pressure-controlled process but a volume-controlled reproduction of the profile. The system demonstrated flexibility to produce combined ventilation patterns by pairing any inspiratory pattern with any expiratory pattern, allowing for customized and complex respiratory profiles. We also implemented specialized patterns for research purposes, such as sinusoidal or rectangular patterns. This flexibility allows the system to interchange different pre-implemented patterns for inspiration and expiration, enabling a wide range of combinations. The system demonstrated efficiency and reliability with minimal boot time. Once the motor is homed, it immediately initiates ventilation with hard-coded standard parameters upon power application (hard reset). A soft reset is implemented in the event of an unexpected system stop, allowing the system to resume ventilation with the last set parameters after homing the stepper motor. We tested various combinations and different timings for inspiration and expiration to achieve the desired tidal volume, demonstrating the system’s functionality, particularly the motor capacities at its fastest velocities and maximal currents.

**Fig. 6:**
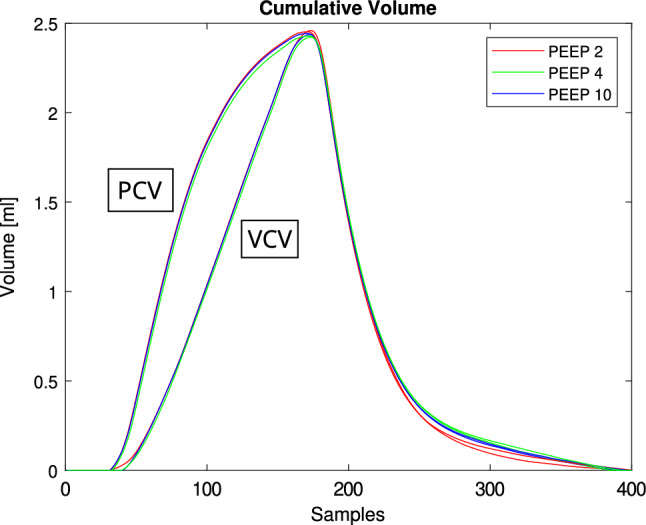
Cumulative volume from flow data for VCV and PCV at varying PEEP levels. This figure presents cumulative volume waveforms derived from flow measurements during ventilation in two modes: volume-controlled ventilation (VCV) and pressure-controlled ventilation (PCV). Each mode was tested under three levels of positive end-expiratory pressure (PEEP): 2, 4, and 10 $\text {cmH}_{2}$O.

**Fig. 7:**
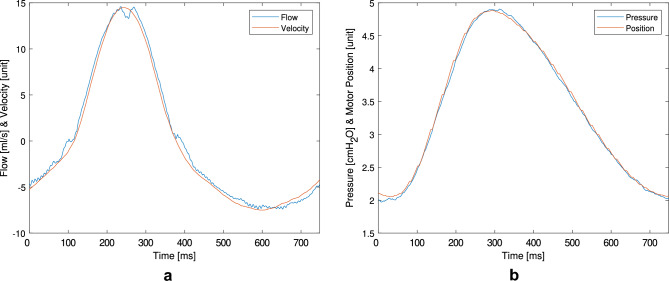
Motor dynamics and respiratory output during sine wave ventilation. This figure compares motor behavior with resulting respiratory parameters during sine wave ventilation. (**a**) Correlation between motor velocity and measured airflow. (**b**) Correlation between motor position and resulting airway pressure.

**Fig. 8:**
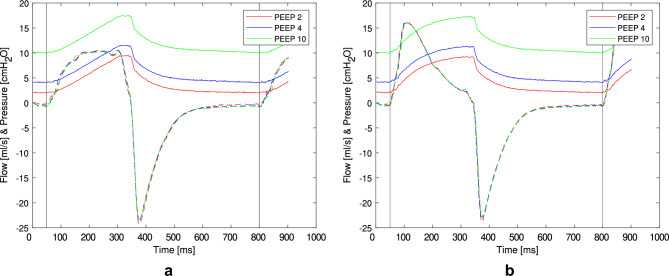
Flow and pressure waveforms for VCV and PCV at three PEEP levels. This figure shows representative flow and pressure waveforms for (**a**) volume-controlled ventilation (VCV) and (**b**) pressure-controlled ventilation (PCV), each tested at three levels of positive end-expiratory pressure (PEEP): 2, 4, and 10 $\text {cmH}_{2}$O.

Flow and pressure values corresponded well with the set parameters on the ventilator. The volume curves calculated from the flow were comparable for different ventilation profiles (PCV and VCV) and remained independent from set PEEP levels (2, 4, and 10 cmH${_2}$O) (Fig. 6). Motor movements were verified, including positions and velocities read from the stepper motor driver, in relation to externally measured flow and pressure. Motor velocity corresponded with flow values, while motor position aligned with pressure measurements, as shown in Fig. 7. Flow and pressure curves were consistent across all common ventilation patterns and various PEEP settings (Fig. 8). Flow values remained constant throughout the breath cycle for every PEEP setting, while pressure values maintained the same pattern but were shifted on the y-axis by the respective PEEP level. PEEP adjustment functioned as intended and could be performed easily. When the PEEP level was reached and the expiratory cylinder withdrew the volume, PEEP was maintained effectively. However, very aggressive expiration patterns, such as exponential expiration combined with high tidal volume, occasionally led to temporary PEEP undershoot.

**Fig. 9:**
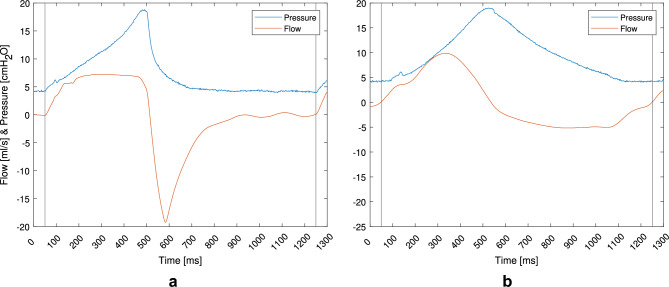
Flow and pressure during rat ventilation with VCV and sinusoidal pattern. This figure presents flow and airway pressure waveforms recorded during in-vivo ventilation of rats using two different inspiratory flow profiles, i.e., (**a**) VCV and (**b**) Sine.

Rats were successfully ventilated using VCV and Sine patterns (Fig. 9). There were no differences in respiratory rates while maintaining paCO${_2}$ between 35 and 45 mmHg. Target tidal volume and PEEP during in-vivo experiments were achieved with high accuracy for both externally measured tidal volume and PEEP. Maximum pressure and driving pressure (defined as the difference between P_max_ and PEEP) were comparable for both ventilation patterns. However, mean pressure was slightly higher for the sine profile (Table 2).

**Table 2:** Ventilation parameters for VCV and Sine profiles during rat ventilation.

	Weight	target V_T_	RR	PEEP	P_max_	P_mean_	P_driving_	V_T_	V_mean_	Max Exp Flow	Max Insp Flow
VCV mean	385.6	3.08	47.53	4.09	17.08	7.11	12.99	3.07	0.94	-23.90	8.25
VCV SD	24.27	0.19	2.33	0.26	2.02	0.38	2.03	0.22	0.19	3.35	0.85
VCV median	389.0	3.11	48.00	4.03	17.45	7.10	13.42	3.09	0.93	-24.97	8.06
Sine mean	385.6	3.08	47.53	4.08	17.40	9.60	13.32	3.03	1.57	-5.81	11.14
Sine SD	24.27	0.19	2.33	0.31	1.79	0.66	1.65	0.22	0.19	0.53	0.91
Sine median	389.0	3.11	48.00	4.07	17.86	9.69	13.82	3.05	1.56	-5.68	11.10

## Discussion

Our self-developed ventilator successfully provided ventilation corresponding to typical ventilation parameters and recommendations reported in other studies^6,16,17^. The system, constructed from off-the-shelf components, demonstrated correct valve functionality and reliable performance.

While the system performs reliably, there is potential for improvement, particularly in the valve components. Although they work effectively in our setup, enhancements could focus on minimizing the impact of valve opening and closing on pressure and flow within the system. Further optimization could be achieved with higher-quality or customized hardware, including specialized valves. However, such customized solutions often result in pattern-limited ventilators, and validated systems can be very expensive and unsuitable for dedicated research applications. A comparison of commonly used commercial ventilators and custom-developed research ventilators is summarized in Table 3. For clarity, only the most advanced or best-performing devices from each manufacturer or author were selected for comparison. All listed commercial devices are currently available for purchase from their respective manufacturers. Estimating the material costs for custom-developed ventilators is challenging due to limited availability of detailed component information in most publications. For comparison, we have listed our previous ventilator^18^, which was entirely built from custom-constructed and self-developed components.

**Table 3:** Comparison of commercial and custom-developed small animal ventilators.

Company/model /author	Ventilation patterns	V_T_ [ml]	RR [$$\text {min}^{-1}$$]	PEEP [$$\text {cmH}_2$$O]	Controlled expiration	I:E	Price
Carfil UMV-03	Sinusoidal	0.1–24	15–220	External	Yes	Fixed 1:1	$4157
Kent Scientific RoVent	PCV, VCV	0.01–12	20–350	0–9	No	1:1 to 1:5	$5016
CWE SAR-1000	PCV, VCV	0.2–30 (max 125)	5–200	No	No	10–90%	$4750
Harvard Apparatus VentElite	PCV, VCV	0.05–5	10–300	0–10	No	20–80%	$6590
RWD R420	PCV, VCV	4–1500	2–60	3–20	No	1:1 to 1:4	$4180
Scireq flexiVent	Various patterns	Depends on module	6–600	0–20	No	2:1 to 1:7	$38,200
Popoiu et al.^17^ (mode 1)	PCV	N/A	20–40	Fixed 3	No	Fixed 1:2	
Popoiu et al.^17^ (mode 2)	PCV	N/A	30–110	Fixed 3	No	Fixed 1:1	
Volgyesi et al.^19^	VCV	0.4–10	Up to 120	External	No	No info	
Schuessler et al.^20^	Various patterns	up to 4	Up to 150	External	No	No info	
Lozano-Zahonero et al.^18^	Various patterns	up to 4.5 (max 9)	Up to 120	0–15	Yes	Variable	
Bonatti et al.^21^	Sinusoidal	up to 3 (max 10)	Up to 200	External	Yes	Variable	
Our ventilator	Various patterns	1–15 (max 35)	Up to 120	0–15	Yes	Variable	$800

Compared to commercially available ventilators, our system provides a highly cost-effective alternative while maintaining support for standard ventilation modes (PCV, VCV) and offering comparable tidal volume ranges (1–15 ml, expandable to 35 ml) and respiratory rates (up to 120 $$\text {min}^{-1}$$). Beyond these standard functions, our system integrates key advanced features such as controlled expiration, recruitment maneuvers, multiple ventilation patterns, and adjustable inspiratory-to-expiratory (I:E) ratios–capabilities that are not universally available in commercial models. Despite the inherent challenges of replicating small animal ventilators with custom-built components, our system represents an accessible and affordable solution for biomedical research, with an estimated cost of approximately $800. This represents a significant cost reduction compared to commercial alternatives, which typically range from $4,000 to over $38,000, making advanced ventilation research more accessible to laboratories.

## Conclusion

We developed and validated a multi-mode small animal ventilator with the aim to significantly enhance small animal research by addressing the limitations of expensive and functionally limited ventilators currently available on the market and in research settings. By thoroughly characterizing the entire system, from the cylinders through the valves and the pipes to the animals’ lungs, we achieved excellent precision in delivering flow, volume, and pressure. Our system enables precise volume delivery with control over even the smallest increments. Additionally, with the manual PEEP setting, we ensure rapid stabilization within a few breaths and maintain consistency throughout the ventilation period. Validation in both in-vitro setups and anesthetized rats confirmed its functional precision, physiological stability, and suitability for preclinical studies. The use of commercially available components allows us to create a low-cost solution that offers a broad range of adjustment options and precise control over ventilation parameters. This enhances both the accuracy of data collected during small animal trials and the reliability of experimental outcomes. The system not only supports research groups conducting animal studies, where animals are under general anesthesia while other non-lung-related experiments are performed, but it also ensures that the animals remain safe throughout the entire duration of the experiment. Furthermore, it grants researchers remarkable flexibility in defining custom ventilation patterns, accommodating any mathematically representable curves and parameters that can be dynamically adjusted according to the characteristics of the system and its individual components.

## Supplementary Information

Supplementary Information.

## Data Availability

The datasets used and/or analyzed during the current study are available from the corresponding author on reasonable request.
