# Supplementary material for: Design and development of an affordable multi-mode small animal ventilator
Source: Sci Rep. 2025 Nov 18;15:40337. doi: 10.1038/s41598-025-28241-w (PMC12627418; doi:10.1038/s41598-025-28241-w)
Supplement: Supplementary file 1 — Supplementary Information. [file 41598_2025_28241_MOESM1_ESM.pdf]

Supplementary Information: Design and Development of an Affordable Multi-Mode Small Animal Ventilator

Patryk Dzierzawski[1,*], Bernd Flamm[1], Verena Hegele[1], Sashko Spassov[1], Christin Wenzel[1], Stefan Schumann[1], and Sara Lozano-Zahonero[1]

[1]Department of Anaesthesiology and Critical Care, Medical Center – University of Freiburg, Faculty of Medicine, Freiburg, 79106, Germany
[*]patryk.dzierzawski@email.uni-freiburg.de

Supplementary Methods

In the supplementary information, we provide detailed technical documentation of the developed ventilator. This includes the complete component list (Supplementary Table S1), a three-dimensional CAD model (Supplementary Figure S1) illustrating the mechanical layout, an electrical circuit diagram (Supplementary Figure S2) depicting the control and sensor connections, and a pneumatic schematic (Supplementary Figure S3) showing the airflow pathways and valve configuration. Together, these resources allow full reproducibility and facilitate adaptation of the system for related experimental setups.

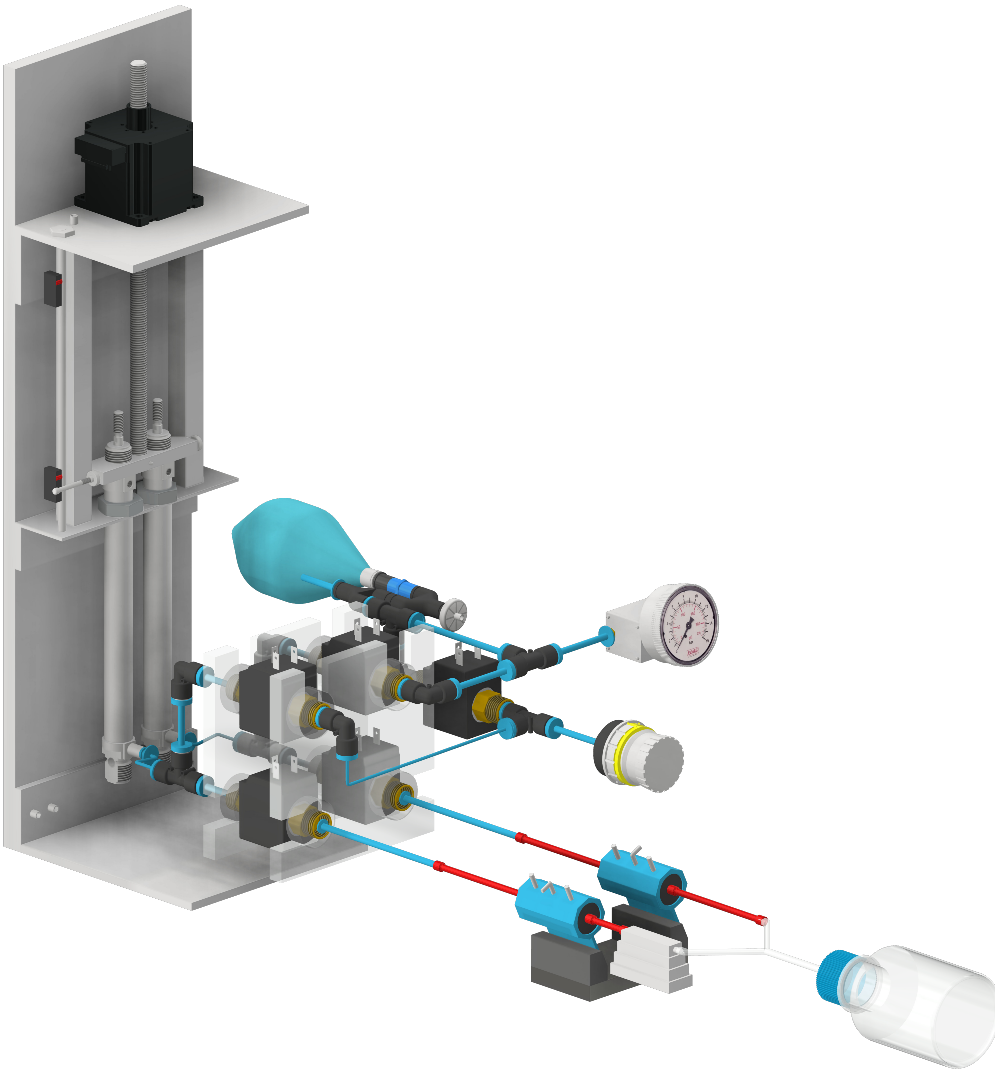

Supplementary Figure S1. CAD model of the designed small animal ventilator.

Supplementary Table S1. Component list

Subsystem	Component	Model / Specification	Manufacturer / Function
**Control electronics**	Microcontroller	Atmel ATmega2560 (Arduino Mega 2560 Rev3)	Atmel / Arduino; main controller for motor and valves
	Programmer / debugger	Microchip Atmel-ICE	Microchip; firmware upload and debugging
	Stepper motor driver	Pololu Tic 36v4	Pololu; UART-based driver for precise stepper control
**Actuation system**	Linear stepper motor	Nanotec LA561S20-B-UQKE	Nanotec; bipolar stepper with lead screw, 25.4 µm per half-step
	Pneumatic cylinders (2×)	Festo DSNU-16-125-PPV-A	Festo; Ø16 mm, 125 mm stroke, ≈25 ml volume each
**Valve system**	Solenoid valves (5×)	BMV70303 (2/2-way, coaxial, 4 mm ID)	Bavaria Fluid Systems; connects and disconnects different airway limbs
	H-Bridge drivers	L298N	STMicroelectronics; bidirectional valve actuation
**Pressure regulation**	PEEP valve	Dräger PEEP Valve	Dräger; 0–15 cmH_2O
	PIP valve	Dräger PIP Valve	Dräger; inspiratory stabilization
**Gas supply & mixing**	Air/O_2 mixer	Siemens-Elema AB system	Siemens-Elema; adjustable FiO_2 21–100%
	Flow limiter / connectors	Siemens-Elema AB system	Siemens-Elema; clinical wall connector and flow restriction
**Sensors & measurement**	Flow sensors	Fleisch 000	Dr. Fenyves & Gut Deutschland GmbH; inspiratory and expiratory flow measurement
	Pressure sensors	Piezoelectric type SI	Si-special instruments GmbH; airway pressure measurement
	Data acquisition (DAQ)	NI 779675-01	National Instruments; 500 Hz sampling, connected to LabVIEW
	Data logging software	LabVIEW	National Instruments; visualization and acquisition of pressure and flow
**Airway circuit**	Tubing	4 mm ID pneumatic tubing	Festo; airflow transport
	Air reservoir	MP02710	Dräger; stabilizes flow
	Exhaust throttle	REF 8402868	Dräger; Provides passive flow resistance

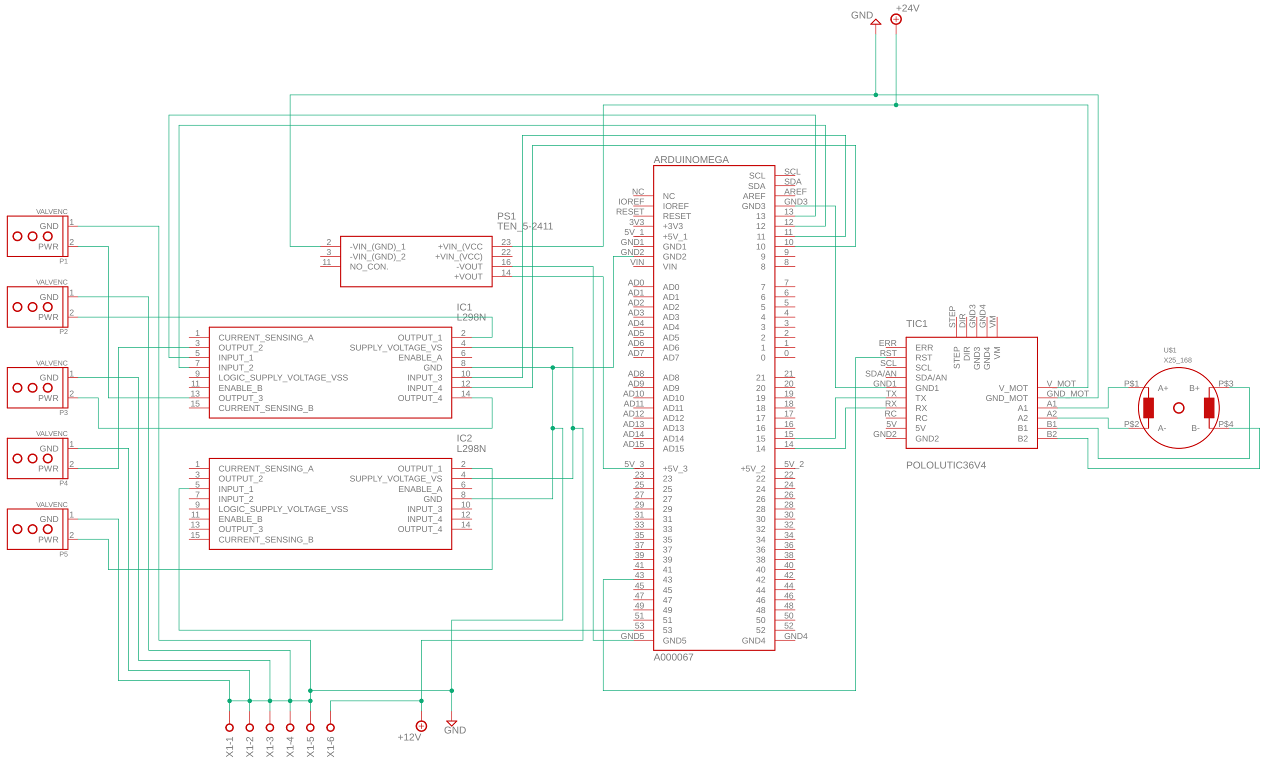

Supplementary Figure S2. Circuit diagram.

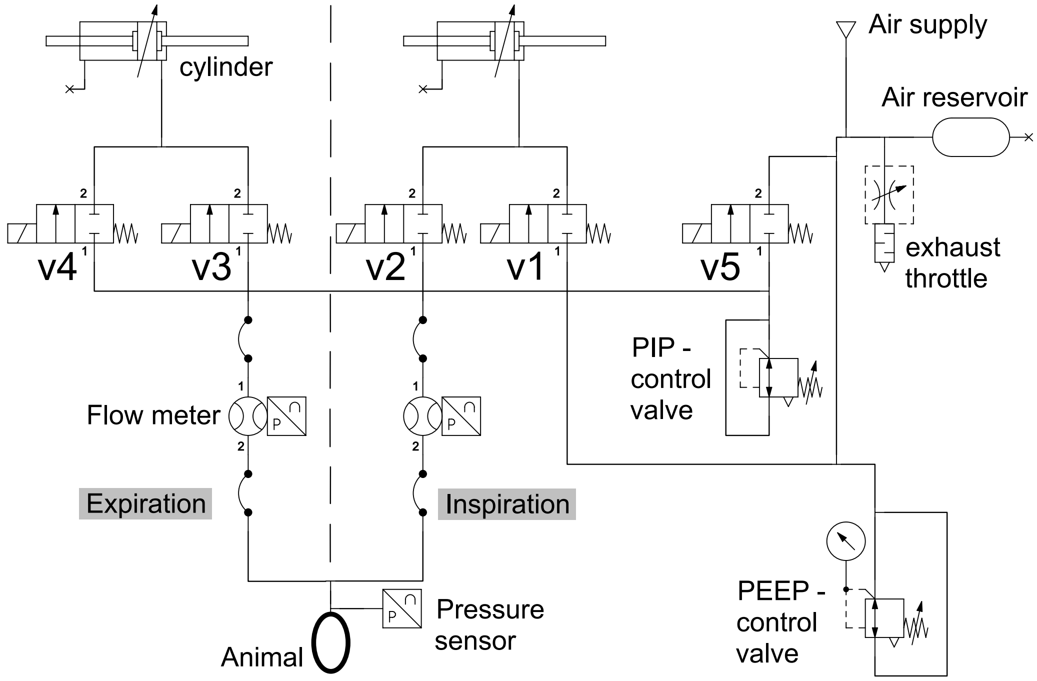

Supplementary Figure S3. Pneumatic schematic of the small animal ventilator.
